# Antitumor Activity of Human Hydatid Cyst Fluid in a Murine Model of Colon Cancer

**DOI:** 10.1155/2013/230176

**Published:** 2013-08-18

**Authors:** Edgardo Berriel, Sofía Russo, Leticia Monin, María Florencia Festari, Nora Berois, Gabriel Fernández, Teresa Freire, Eduardo Osinaga

**Affiliations:** ^1^Laboratorio de Glicobiología e Inmunología Tumoral, Institut Pasteur de Montevideo, 11400 Montevideo, Uruguay; ^2^Clínica Quirúrgica 1, Hospital Pasteur, Facultad de Medicina, Universidad de la República, 11400 Montevideo, Uruguay; ^3^Departamento de Inmunobiología, Facultad de Medicina, Universidad de la República, 11400 Montevideo, Uruguay; ^4^Unidad de Animales Transgénicos y Experimentación (UATE), Institut Pasteur de Montevideo, 11400 Montevideo, Uruguay

## Abstract

This study evaluates the antitumor immune response induced by human hydatic cyst fluid (HCF) in an animal model of colon carcinoma. We found that anti-HCF antibodies were able to identify cell surface and intracellular antigens in CT26 colon cancer cells. In prophylactic tumor challenge experiments, HCF vaccination was found to be protective against tumor formation for 40% of the mice (*P* = 0.01). In the therapeutic setting, HCF vaccination induced tumor regression in 40% of vaccinated mice (*P* = 0.05). This vaccination generated memory immune responses that protected surviving mice from tumor rechallenge, implicating the development of an adaptive immune response in this process. We performed a proteomic analysis of CT26 antigens recognized by anti-HCF antibodies to analyze the immune cross-reactivity between *E. granulosus* (HCF) and CT26 colon cancer cells. We identified two proteins: mortalin and creatine kinase M-type. Interestingly, CT26 mortalin displays 60% homology with *E. granulosus* hsp70. In conclusion, our data demonstrate the capacity of HCF vaccination to induce antitumor immunity which protects from tumor growth in an animal model. This new antitumor strategy could open new horizons in the development of highly immunogenic anticancer vaccines.

## 1. Introduction

Several infectious agents (e.g., the bacterium *Helicobacter pylori*, the human papilloma viruses, and the hepatitis B and C viruses) are considered to be causes of cancer in humans [1]. Pathogens are responsible for about 2 million cases of cancer (16.1%) each year [2]. Among parasites, a carcinogenic role is recognized for *Schistosoma haematobium, Clonorchis sinensis, *and* Opisthorchis viverrini* [3, 4]. Carcinogenesis associated with helminth infections is a complex process, which may involve several different mechanisms, being chronic inflammation a key feature [5]. Contrastingly, the ability of various infective agents to suppress cancer growth has been well documented both in humans [6, 7] and in experimental animal models. A low level of colon cancer induced by 1,2-dimethylhydrazine has been reported in rats chronically infected with *T. cruzi* [8]. In addition, it was also found that malaria infection inhibited Lewis lung cancer growth and metastasis and prolonged the survival of tumor-bearing mice [9].

*Echinococcus granulosus* is a cestode parasite which causes the disease cystic echinococcosis. Regarding *E. granulosus* infection, a significantly lower prevalence of cancer in patients with hydatid disease was reported in a large retrospective study performed by Akgül et al. [10]. van Knapen [11] evidenced antigenic similarities between *E. granulosus* and some tumour types. It is of interest that cancer-associated mucin-type *O*-glycan antigens (such as Tn, TF, and sialyl-Tn) are expressed by some helminth parasites [12]. In line with these results, we found the presence of Tn antigen in larval and adult tissues of *E. granulosus* [13]. Based on these observations we are tempted to hypothesize that certain *E. granulosus* antigens could be involved in the induction of a cross-reactive immune response which would be effective against cancer growth. We present here results evidencing anti-tumor activity of *E. granulosus* by both prophylactic and therapeutic vaccinations. We found that immunization with human hydatic cyst fluid (HCF) induces antibodies against CT26 colon carcinoma cells and protects against tumor growth in mice.

## 2. Material and Methods

### 2.1. Animals and Tumor Cell Line

BALB/c mice were purchased from the Jackson Laboratory (Bar Harbor, ME, USA) and breeded and maintained at the animal facility of Institut Pasteur de Montevideo (Uruguay) under specific pathogen-free conditions. Rabbits were purchased from Instituto de Higiene (Facultad de Medicina, Montevideo, Uruguay). All the animal protocols were approved by Institutional Animal Care Committee and were performed following facility guidelines. The murine colon carcinoma cell line CT26 was obtained from ATCC (Manassas, VA, USA) and was cultured in DMEM (Invitrogen, Carlsbad, CA, USA) supplemented with 10% FBS (Invitrogen) at 37°C temperature and 5% CO_2_ atmosphere.

### 2.2. Hydatid Cyst Fluid

The starting material consisted of three noncomplicated *E. granulosus* hydatid cysts (two localized in the liver and one in the spleen), obtained from patients operated in the Hospital Pasteur, Montevideo, Uruguay. The study was examined and approved by the ethical review board of the School of Medicine, Montevideo, Uruguay. The HCF was aspirated aseptically from fertile cysts then centrifuged at 10000 ×g at 4°C for 30 min, and the supernatant was kept at −20°C until use. The present work was carried out using a batch comprising a pool of the three individual cysts.

### 2.3. Evaluation of Sera Reactivity by Flow Cytometry

Mice or rabbits were immunized three times with human HCF (100 *μ*g protein) in aluminum hydroxide (alum) at two-week intervals. After the last immunization, animals were bled, and sera were evaluated by flow cytometry on the CT26 cell line. Cells were first incubated for 15 min with sera (diluted 1 : 100) at 4°C in PBS containing 2% fetal bovine serum and 0.1% sodium azide. Then, they were incubated for 15 min with an anti-mouse or anti-rabbit IgG goat antibody conjugated to FITC (Sigma, St. Louis, MO, USA). Alternatively, in order to evaluate sera recognition of intracellular antigens, cells were first permeabilized by incubating them in PBS containing 0.1% Triton-X100, 2% fetal bovine serum, and 0.1% sodium azide. Paraformaldehyde-fixed cells were analyzed on a CyAn ADP analyzer (Beckman Coulter), and analyses were performed with Summit V4.3 (Dako).

### 2.4. Two-Dimensional Electrophoresis and Western Blotting

Lysates of CT26 cells were obtained by incubation in lysis buffer (30 mM Tris, 2 M thiourea, 7 M urea, 4% CHAPS, and pH 8.5 with protease inhibitors) at room temperature for 30 min. The sample was centrifuged at 14,000 rpm for 10 min, and the supernatant was used for two-dimensional electrophoresis (2DE). Sample proteins were separated according to their isoelectric point, using strips of 7 cm nonlinear pH range of 3–11 (Immobiline DryStrips, GE Healthcare). For this, 200 *μ*g of CT26 protein lysate was diluted in rehydration solution (8 M urea, 0.5% CHAPS, 0.3% DTT, 0.5% IPG buffer, and 0.002% bromophenol blue). Isoelectric focusing was performed using the IPGPhor (Amersham Bioscience), following the protocol recommended by the manufacturer. SDS-polyacrylamide gel electrophoresis (12.5%) was performed after IPG strip was isoelectrically focused. After 2DE separation, one gel was stained with Coomassie Brilliant Blue, and the other was detected by western blotting. Briefly, the membrane was incubated with a dilution of rabbit anti-HCF serum for 1 hour then washed and incubated with an appropriated dilution of peroxidase conjugated anti-rabbit polyvalent antibodies (Dako) for another hour. The membrane was washed, and spots were developed using ECL Western Blotting Detection Reagents (GE Healthcare). Rabbit preimmune serum was used as control. The spots of the proteins of interest were excised from the gel and analyzed with a 4800 MALDI TOF/TOF analyzer mass spectrometer, operated in reflector mode. The peptide map obtained for each sample was compared with the nonredundant database of known protein tryptic digests of SwissProt (http://us.expasy.org/) or NCBI (http://www.ncbi.nlm.nih.gov/) using the online tool of MASCOT program (http://www.matrixscience.com/).

### 2.5. Mice Tumors and Immunization Strategies

BALB/cJ female mice 6–8 weeks old were injected s.c. into the right flank with 1 × 10^5^ CT26 cells diluted in PBS. In prophylactic experiments, mice were vaccinated three times (days 35, 21, and 7 before tumor cell challenge) with HCF (300 *μ*g protein/mouse) in alum. In the therapeutic setting, mice were challenged on day 0 with 1 × 10^5^ CT26 cells, and 4, 7, and 10 days later they were vaccinated with HCF in alum. Control mice were treated with PBS in alum. The size of the tumor was calculated by the formula *V* (mm^3^) = (4/3) × pi × *R*_1_ × *R*_2_ × *R*_3_, where *R*_1_, *R*_2_, and *R*_3_ are the largest radii of the tumor in three dimensions. Mice were euthanized when the tumor diameter reached 20 mm or if they showed signs of distress. Survival of mice was followed for 90 days.

### 2.6. Statistical Analysis

Student's *t* test was used to compare data from various experimental groups. A *P* value <0.05 was considered statistically significant. Mean and SD are shown unless indicated otherwise. Survival was evaluated from the day of tumor injection until euthanasia, and the Kaplan-Meier test was used to compare mouse survival between the groups. All results are presented as means ± SD. Data were processed using the IBM SPSS Statistics 20.0 software.

## 3. Results

### 3.1. Preventive Vaccination with Human HCF Protects against Tumor Challenge and Rechallenge

**Figure 1:**
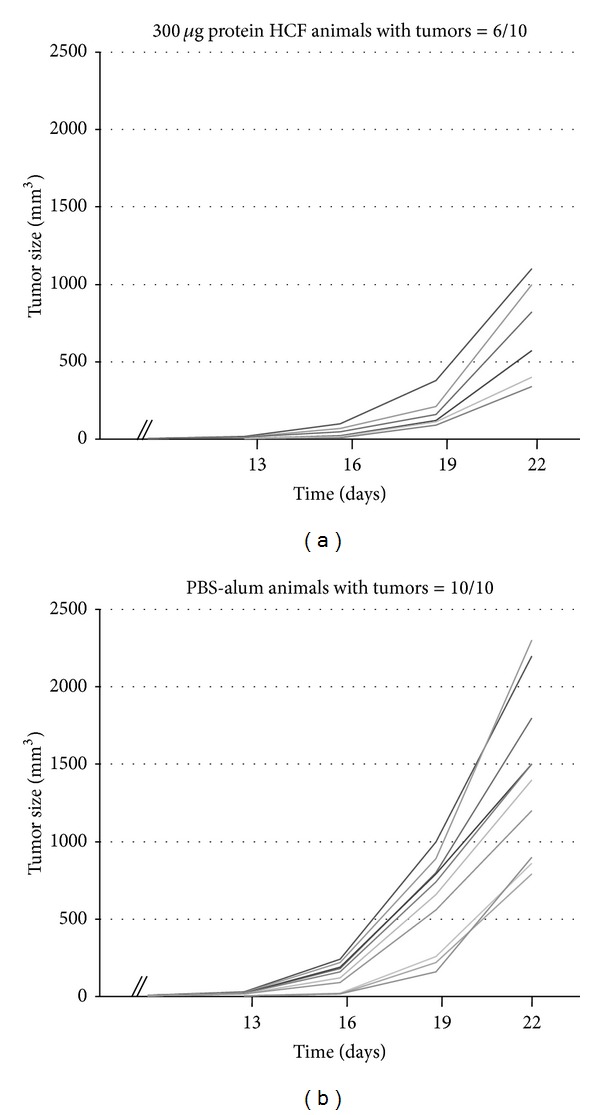
HCF immunization protects against CT26 tumor growth. (a) BALB/cJ mice (*n* = 10) were vaccinated three times in two-week intervals with human HCF in alum before CT26 cells challenge. (b) Control mice (*n* = 10) were treated with PBS in alum. Tumor growth was measured regularly using a caliper. Tumor volume (mm^3^) = (4/3) × pi × *R*_1_ × *R*_2_ × *R*_3_. Tumor sizes were significantly lower in mice immunized with HCF as compared to the control group (*P* = 0.006).

**Figure 2:**
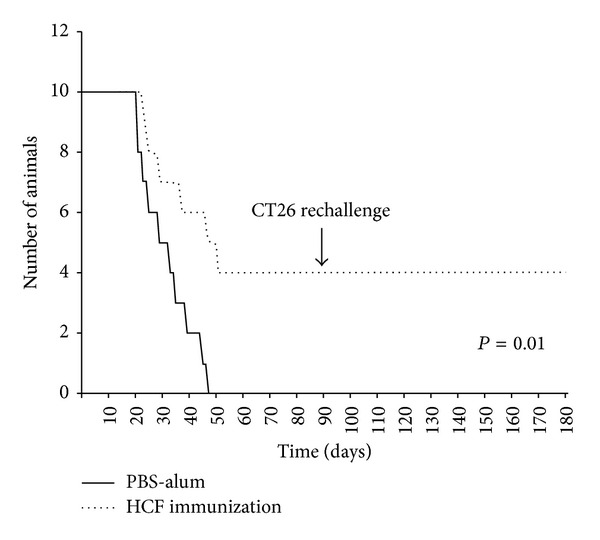
HCF prophylactic vaccination improved the survival in tumor-bearing mice. Survival of treated and control mice was followed for 100 days after tumor challenge. Mice were euthanized when subcutaneous tumors reached 20 mm or when mice became moribund. Tumor-free surviving mice (*n* = 4) previously treated with HCF were rechallenged with CT26 cells at day 90. Representative results of one of 3 independent experiments are shown.

In prophylactic studies, 7 days after the last boost, mice were challenged with 1 × 10^5^ CT26 cells, and survival of mice was followed for 90 days. First, we compared the antitumor activity of HCF at different concentrations of immunogen (75 *μ*g, 150 *μ*g, 300 *μ*g, and 600 *μ*g proteins) observing that the 300 *μ*g protein dose generated the higher protection against tumor challenge (data not shown). Consequently, this concentration was used in subsequent experiments. The average tumor size was significantly lower (*P* = 0.006) in mice immunized with HCF as compared to the control group (PBS-alum) (Figure 1). All mice treated with PBS-alum were euthanized within 48 days following tumor challenge (Figure 2). In contrast, 40% of mice vaccinated with HCF-alum survived without tumor burden by the end of the experiment period (*P* = 0.01). 

Mice that survived without tumor burden in prophylactic experiments were rechallenged with 1 × 10^5^ CT26 colon cancer cells 90 days after the first tumor inoculation. As controls, naïve mice were also injected with the tumor cells. All four mice receiving the HCF-alum vaccine survived without detectable tumor burden after tumor rechallenge (they were still tumor free 3 months later) (Figure 2), while all control mice had to be euthanized within 50 days from tumor injection (data not shown). These results suggest that human HCF may also induce antigen specific immunologic memory against CT26 colon cancer cells.

### 3.2. Immunotherapeutic Vaccination with HCF Increases Mouse Survival

**Figure 3:**
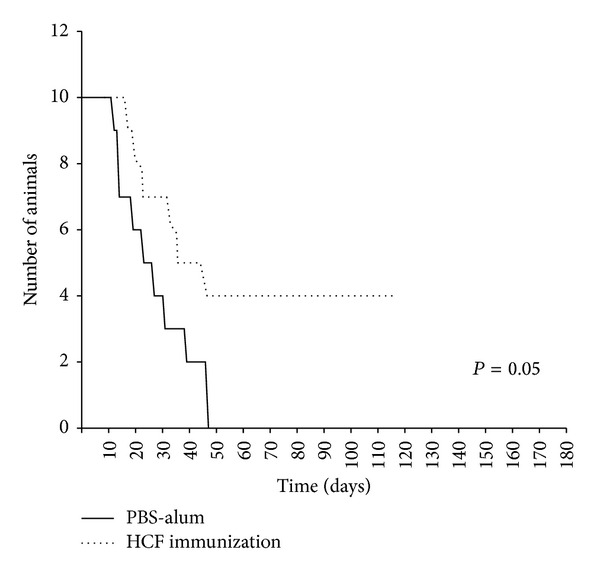
HCF-based active immunotherapy improved the survival in tumor-bearing mice. After s.c. administration of CT26 colon cancer cells (day 0), 6-week-old BALB/c mice received a 300 *μ*g dose of human HCF in alum on days 4, 7, and 11. Control animals received PBS in alum. Mice were monitored for survival as described in Figure 2(b). Mice survival was followed for 100 days after tumor challenge. Representative results of one of 2 independent experiments are shown.

We next evaluated the efficacy of HCF to induce protection against tumor growth in a therapeutic setting. To this end, mice were inoculated with 1 × 10^5^ CT26 cells and were then treated at days 4, 7, and 11 with 300 *μ*g of HCF in adjuvant (aluminum hydroxide) or with adjuvant alone (control group). In these conditions, the most remarkable finding was the survival of 40% HCF-treated mice, whereas all control mice were euthanized (Figure 3). These differences were statistically significant (*P* = 0.05). 

### 3.3. Human HCF Induces Antibodies That Recognize CT26 Colon Cancer Cells

**Figure 4:**
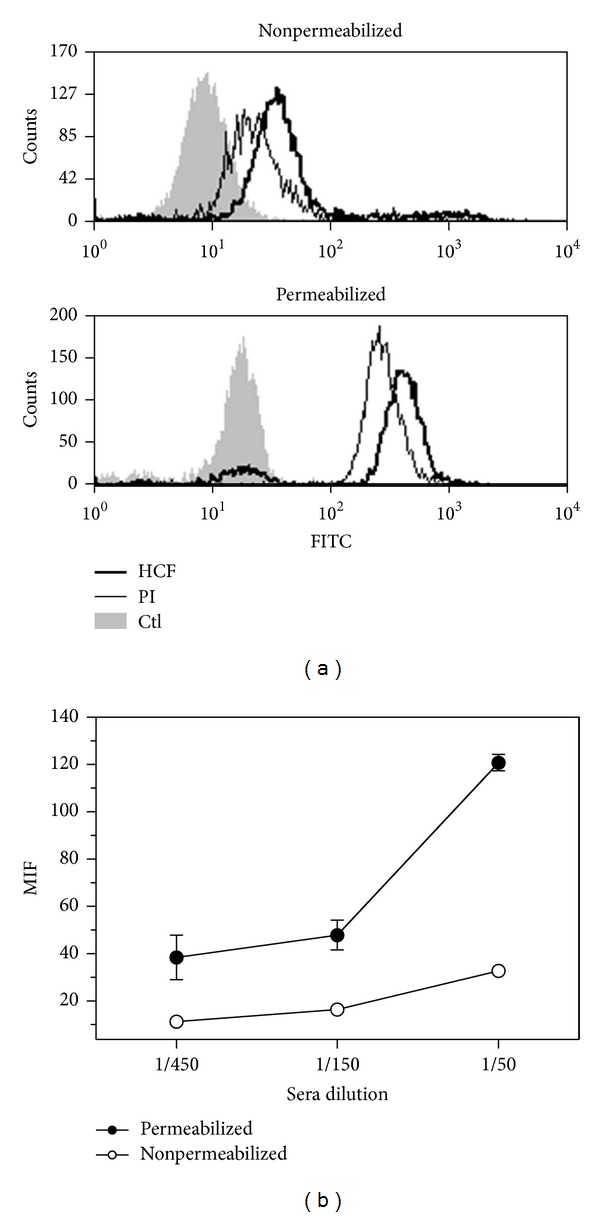
Recognition of CT26 cells by sera from HCF-immunized animals. (a) Histogram plots showing antibody recognition for membrane and cytosolic antigens on CT26 cells. Flow cytometry analyses were carried out on permeabilized or nonpermeablized CT26 tumor cells incubated with sera (diluted 1 : 100) collected from animals immunized with human HCF. Controls consisted of preimmune sera (PI) or cells incubated with secondary antibody only (Ctl). Five thousand events were collected and gated on FSC versus SSC dot plot. (b) Median fluorescence intensity (MFI) representing antibody recognition of membrane and cytosolic antigens on nonpermeabilized and permeabilized CT26 cells, respectively. In this case, different sera dilutions (1 : 50, 1 : 150, and 1 : 450) were used, and MFI values were subtracted to the corresponding preimmune sera at the same dilution.

Considering that our results strongly suggested the involvement of adaptive immunity in the anti-tumor response induced by HCF, we evaluated whether mice immunized with HCF developed specific antibodies capable of recognizing tumor cells. Flow cytometric analyses of the CT26 cells indicated that this cell line was recognized by HCF-induced antibodies. Indeed, an antihuman HCF serum was able to bind cell surface antigens as well as intracellular antigens in most CT26 cells (Figure 4(a)). This recognition pattern was confirmed at different serum dilutions (Figure 4(b)). Taken together, these results indicate that anti-HCF antibodies cross-react with molecules expressed on CT26 cells.

### 3.4. Proteomic Analysis of CT26 Antigens Identified by Anti-HCF Antibodies

**Figure 5:**
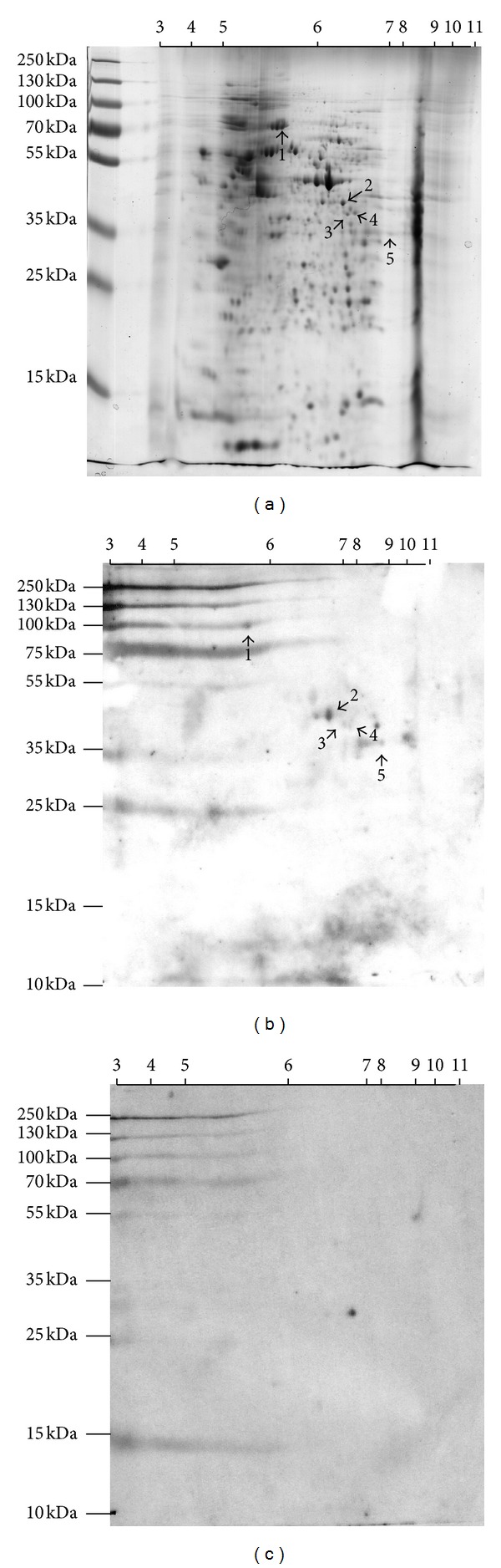
Proteomic analysis of CT26 antigens identified by anti-HCF antibodies. 2DE was performed with 200 *μ*g protein using strips of 7 cm nonlinear pH range of 3–11 and 12.5% SDS-PAGE. (a) Gel was stained with Coomassie Brilliant Blue. (b) Western blot using anti-HCF serum. (c) Western blot using preimmune serum. By mass spectroscopy, spot 1 was identified as mortalin, whereas spot 3 was identified as creatine kinase M-type. Spots 2, 4, and 5 were not identified using the tool online of MASCOT program (http://www.matrixscience.com/).

Next, we carried out a proteome-based approach in order to identify CT26 antigens recognized by anti-HCF antibodies. CT26 proteins were separated by 2DE, and the gel was stained with Coomassie Brilliant Blue (Figure 5(a)). Subsequently, CT26 proteins separated by 2-DE were analyzed by western blotting using an anti-HCF serum (Figure 5(b)). Nonspecific recognition by the serum was identified by the use of a preimmune serum (Figure 5(c)). We found 5 protein spots specifically identified by the anti-HCF serum. These proteins were analyzed by MALDI TOF/TOF-MS, and two of them were identified as mortalin [14] and creatine kinase M-type (EC = 2.7.3.2). Exploring the *E. granulosus* nonredundant protein sequences data base (blastp, protein-protein BLAST), we did not found any significant homology between creatine kinase M-type and *E. granulosus* proteins. However, mouse mortalin displays 60% homology with *E. granulosus* hsp70. Mortalin (mitochondrial hsp70) was first cloned as a novel member of the hsp70 family of proteins from the cytoplasmic fractions of normal fibroblasts [14]. This protein is overexpressed in tumor cells and binds to p53 protein. Several observations have suggested that mortalin is involved in the transformation of normal cells to cancer cells [15], in a process that involves mortalin interaction with p53 promoting sequestration of p53 in the cytoplasm, thereby inhibiting its nuclear activity [16].

## 4. Discussion

Current cancer immunotherapy strategies target cancer cells directly or indirectly via generation of host immune cell responses to tumor associated antigens (TAA) [17]. Cancer vaccination is an important and promising approach in cancer immunotherapy. Obstacles for clinical success may include immune tolerance to TAAs, the weak antigenic nature of TAAs, and active immune evasion mechanisms employed by progressing tumors [18]. Successful vaccine formulations may require a nontoxic immunomodulator or adjuvant that not only stimulates innate and adaptive tumor-specific immune responses but also overcomes immune evasion mechanisms [19]. Vaccination with TAAs coming from evolutionary distant organisms (such as *E. granulosus*) should be useful to override tolerance problems encountered with human TAA-based cancer therapeutic approaches [20]. The goal of our study was to examine whether HCF from patients with hydatid disease could be used as a tumor vaccine to elicit CT26-specific immunity. HCF immunization was able to induce antibodies that recognized CT26 colon carcinoma cells and to prevent tumor growth. To our knowledge, this study represents the first successful attempt to induce an effective anti-tumor immune response using HCF that can control cancer growth *in vivo*. This vaccination generated immunological memory that protected surviving mice from tumor rechallenge, indicating the participation of adaptive immunity. 

Certain parasite products, including hydatid cyst protoscolices [21], are able to inhibit tumor growth [22–24], suggesting that these parasites may have anti-tumor properties. In addition, it has been demonstrated that the anti-cancer activity of some parasites is mediated by the induction of anti-tumor immunity. Chen et al. [9] found that malaria infection significantly suppresses Lewis lung cancer growth via induction of innate and specific adaptive anti-tumor responses with production of T helper 1 (Th1) cytokines. More recently, it was reported that an intratumoral injection of a live attenuated strain of *Toxoplasma gondii* stimulated anti-tumor immune responses *in vivo* that regressed established primary melanoma B16F10 murine tumors [25]. Several hypotheses may explain the anti-CT26 tumor immunity afforded by the HCF treatment. For instance, the putative anti-tumor activity of anti-CT26 antibodies induced by HCF is supported by the observation that sera from patients with hydatid cysts had a lethal effect on human small cell lung cancer cells *in vitro* [26]. It has been also found that HCF elicits both Th1 and Th2 cell activations [27, 28]. Th1 cell activation is related to protective anticancer immunity. In addition, HCF can stimulate predifferentiated dendritic cells to mature, as evidenced by release of IL-6 and IL-12 and by up-regulation of class II major histocompatibility complex and CD86 [29]. 

There are several candidate molecules in HCF that could act as antigens or adjuvant components. The antigenic signatures that characterize *E. granulosus* HCF are antigen 5 [30] and antigen B [31]. In our attempt to identify proteins involved in the cross-reactivity between CT26 cancer cells and *E. granulosus*, we found that creatine kinase M-type and mortalin were recognized by anti-HCF antibodies. Mortalin overexpression was associated with a more aggressive biology and poor prognosis in colorectal cancer patients [32]. Mortalin is a member of hsp70 family which presents 60% homology compared with hsp70 from *E. granulosus*. Many different functions have been attributed to hsp70 depending on its location. Intracellular hsp70 allows the cells to survive potentially lethal conditions, explained by its antiapoptotic properties. On the other hand, extracellular or membrane-bound hsp70 mediates some immunological functions, such as eliciting an anti-tumor response that provides a link between innate and adaptive immunity. Due to hsp70 chaperone activity, hsp70-tumoral peptides can interact with dendritic cells through different receptors. After endocytosis, the complexes are degraded, and tumoral peptide could be cross-presented to CD8 T cells [33]. Hsp70-based vaccines can activate tumor-specific immunity, inhibiting tumor growth [34]. It was previously shown that hsp70 is an immunodominant antigen in echinococcal disease [35], and that it is able to induce both B and T cell responses [36]. Taken together, *E. granulosus* HCF may be a good vaccine vehicle not only for presentation of tumor-associated determinants but also for its adjuvant properties (such as hsp70) that provide the appropriate milieu to enhance the efficacy of antigen presentation to dendritic cells.

In conclusion, we report here that human HCF immunization significantly inhibits colon cancer growth via induction of antitumor immunity. Although our results suggest that anti-HCF antibodies may participate in the anti-tumor effect, a thorough characterization of the immune processes responsible for tumor rejection is necessary. In order to determine whether immunization with *E. granulosus* antigens could be the basis for a new type of anti-tumor vaccine, we will expand our results using HCF immunization in other animal cancer models.
